# Combining Crop Growth Modeling With Trait-Assisted Prediction Improved the Prediction of Genotype by Environment Interactions

**DOI:** 10.3389/fpls.2020.00827

**Published:** 2020-06-19

**Authors:** Pauline Robert, Jacques Le Gouis, The BreedWheat Consortium, Renaud Rincent

**Affiliations:** INRAE, UCA, UMR 1095 Génétique, Diversité et Ecophysiologie des Céréales, Clermont-Ferrand, France

**Keywords:** crop growth model, gene-based modeling, genomic selection, genotype × environment interaction, multi-environment trials, wheat

## Abstract

Plant breeders evaluate their selection candidates in multi-environment trials to estimate their performance in contrasted environments. The number of genotype/environment combinations that can be evaluated is strongly constrained by phenotyping costs and by the necessity to limit the evaluation to a few years. Genomic prediction models taking the genotype by environment interactions (GEI) into account can help breeders identify combination of (possibly unphenotyped) genotypes and target environments optimizing the traits under selection. We propose a new prediction approach in which a secondary trait available on both the calibration and the test sets is introduced as an environment specific covariate in the prediction model (trait-assisted prediction, TAP). The originality of this approach is that the phenotyping of the test set for the secondary trait is replaced by crop-growth model (CGM) predictions. So there is no need to sow and phenotype the test set in each environment which is a clear advantage over the classical trait-assisted prediction models. The interest of this approach, called CGM-TAP, is highest if the secondary trait is easy to predict with CGM and strongly related to the target trait in each environment (and thus capturing GEI). We tested CGM-TAP on bread wheat with heading date as secondary trait and grain yield as target trait. Simple CGM-TAP model with a linear effect of heading date resulted in high predictive abilities in three prediction scenarios (sparse testing, or prediction of new genotypes or of new environments). It increased predictive abilities of all reference GEI models, even those involving sophisticated environmental covariates.

## Introduction

The objective of plant breeders is to develop varieties well adapted to target environments. For this purpose, they evaluate each year candidate varieties in multi-environment trials (MET). Given that the speed of the process is a key element of genetic progress and since phenotyping is expensive, most varieties are evaluated in a small number of environments, considered as a combination of year × site × condition. Consequently, the environments in which the varieties are evaluated can be quite different from the target environments, because of the significant variation between years. In addition, only a limited number of varieties are evaluated each year to control the phenotyping costs. All these constraints are reducing the chance of success as they limit the number of lines that can be evaluated in the target environments.

One way to raise this strong constraint is to predict the performance of candidate varieties using molecular information with genomic selection (GS) models (Whittaker et al., 2000; Meuwissen et al., 2001). In GS, a phenotyped and genotyped calibration set is used to estimate DNA marker effects. Once the model is calibrated, new candidate varieties can be predicted, as long as their genotypic information is available. Specific GS models were proposed to predict the performance of varieties in different environments, taking the genotype by environment interaction (GEI) into account. It was first proposed to adapt the reference GS models to the GEI context by attributing environment specific effects to the markers (Schulz-Streeck et al., 2013; Crossa et al., 2016), or by modeling genetic covariances between environments (Burgueño et al., 2012). In other studies, environmental covariates (EC) were introduced in the GS model (Heslot et al., 2014; Jarquín et al., 2014; Malosetti et al., 2016; Ly et al., 2018), which allows predicting the performance of varieties in new environments. Crop growth models (CGM) were sometimes used to adjust the EC estimates to phenological stages, or to derive EC estimating the stress experienced by the plants (Ly et al., 2017; Rincent et al., 2019), instead of directly using pedoclimatic data.

One efficient way to improve the prediction accuracy of GEI is to introduce secondary traits measured in each environment on both the calibration set and the test set in multi-trait GS models. It was indeed shown that if the secondary trait is sufficiently heritable and correlated to the target trait, the approach called “trait-assisted prediction” (TAP) or “phenotype imputation”, can be very efficient (Henderson and Quaas, 1976; Calus and Veerkamp, 2011; Jia and Jannink, 2012; Pszczola et al., 2013; Fernandes et al., 2018; Bustos-Korts et al., 2019). It resulted in successful applications in wheat (Rutkoski et al., 2016; Sun et al., 2017, 2019; Crain et al., 2018; Lado et al., 2018; Michel et al., 2018; Schulthess et al., 2018). In particular Rutkoski et al. (2016) showed that secondary physiological traits measured in each environment on both predicted and test sets resulted in increased predictive ability for grain yield using a TAP model. TAP is particularly useful when the secondary trait is easy to obtain, highly heritable and strongly correlated to the target trait. In MET, the secondary trait measured in each environment can capture GEI and serves as a proxy of the target trait in each environment (Bustos-Korts et al., 2019). But the applicability of this approach in the GEI context is considerably constrained by the necessity to phenotype all varieties (calibration and test sets) in all environments for the secondary trait, which means that the test set has to be sown and phenotyped in each environment. The interest of this approach would be considerably stronger if the secondary trait would be accessible without phenotyping the test set.

Crop Growth Models (CGM) are also powerful tools to predict GEI. They model plant development using mechanistic relationships with genetic characteristics (genetic parameters) and environmental variables as input. These genetic parameters characterize the varieties (e.g., sensitivity to photoperiod) independently from the environment, and so are supposed to be constant from one environment to another for a given variety (Reymond et al., 2003, 2004). A method combining CGM and GS, called gene-based modeling (GBM), can be used to predict GEI for unobserved varieties. If the genetic parameters of varieties of the calibration set are known, a GS model can be calibrated and used to predict the genetic parameters of the test varieties. These predictions can then be used as input for the CGM together with environmental variables to predict the performance of the test set in various environments.

This strategy has proven to be effective on simple traits such as leaf elongation rate in maize (Reymond et al., 2003; Chenu et al., 2008), fruit quality (Quilot et al., 2005; Prudent et al., 2011), and phenology of various species (White and Hoogenboom, 1996; Nakagawa et al., 2005; Yin, 2005; Messina et al., 2006; White et al., 2008; Uptmoor et al., 2011; Zheng et al., 2013; Onogi et al., 2016) including wheat (Bogard et al., 2014; Rincent et al., 2017). The major limit of this approach is that it remains difficult to efficiently predict complex traits such as yield, although promising results have been obtained (Technow et al., 2015; Cooper et al., 2016; Messina et al., 2018). In any case, GBM can be very efficient to predict secondary traits, for example related to phenology. If we were able to use CGM (or GBM for new varieties) to accurately predict a secondary trait correlated to the final target trait in each environment, then we would be able to combine CGM and trait assisted prediction to overcome the limits of both approaches. The idea here is to use a TAP approach in which the secondary trait is not phenotyped for the test set (so there is no need to sow the test set in any experiment) but predicted in each environment of interest thanks to CGM.

We propose to test this new approach (CGM-TAP) on winter wheat with grain yield (GY) as target trait (the trait the breeder is interested in), and heading date (HD) as secondary trait. HD is very highly correlated to flowering time, which is indeed an important adaptive trait with an optimal date depending on the environment. Phenology is a major trait for the plants to benefit from the most possible resources while avoiding stressing conditions at key stages (Richards, 1991; Lopes et al., 2014; Semenov et al., 2014; Flohr et al., 2017). In addition, it was shown that GBM was efficient to predict HD in winter wheat (Bogard et al., 2014; Rincent et al., 2017). Therefore, this trait seems to be a particularly good candidate to test the CGM-TAP approach with GY as target trait. For this purpose, we used a MET composed of 220 varieties and 42 managed environments, that was used to compare the accuracy of CGM-TAP to reference GEI models in different prediction scenarios: the prediction of new varieties, of new environments, or of the missing phenotypes of an incomplete design (sparse testing).

## Materials and Methods

### Genetic Material, Genotyping, and Estimation of a Genetic Covariance Matrix (Kinship)

The plant material has been previously described in Rincent et al. (2018, 2019) and Touzy et al. (2019). The genetic panel is composed of 220 European elite varieties of winter wheat. It was genotyped with the TaBW280K high-throughput genotyping array described in Rimbert et al. (2018). This array was designed to cover both genic and intergenic regions of the three bread wheat subgenomes. Markers with a minor allele frequency (MAF) below 5%, or with heterozygosity or missing data rate above 5% were removed. Markers in strong Linkage Disequilibrium (LD) were filtered out using the pruning function of Plink (Purcell et al., 2007) with a window of size 100 SNPs, a step of 5 SNPs and a LD threshold of 0.8, as proposed in Charmet et al. (2020). Eventually, we obtained 26,116 polymorphic high resolution SNPs, with an average missing data rate of 1.0%. Missing values were imputed as the marker observed frequency.

Genotype of individual *i* at marker *l* (*M*_*i,l*_) was coded as 1, 0.5, or 0 for homozygote for an arbitrarily chosen allele, heterozygote, and the other homozygote, respectively. Genomic relatedness (kinship) between individuals was estimated following (VanRaden, 2008):

$K_{i,j}=\sum_{l=1}^{L}\frac{\left(M_{i,l}-p_{l}\right)\,\left(M_{j,l}-p_{l}\right)}{\text{b}}$, with $b=\sum_{l=1}^{L}p_{l}\times \left(1-p_{l}\right)$, *p*_*l*_ being the allelic frequency of the reference allele in the corresponding diversity panel, *L* the number of markers. The matrix of genomic relatedness coefficients will be denoted *K* in the manuscript.

### Phenotypic Data Measured in the Multi-Environment Trial

The same phenotypes as in Rincent et al. (2019) were used. Briefly, the panel was phenotyped for grain yield (GY) and heading date (HD) in a multi-environment trial composed of 42 managed environments located in France between 2012 and 2016 (Supplementary Table S1). These 42 managed environments correspond to 26 combinations of years and locations, with two treatments for 16 of them. Among these 16 combinations of years and locations, three had an irrigated (WW) and a rainfed (WD) treatment, one had a well-watered (WW) and a rainout shelter (RO) treatments, and 12 had a high (HN) and a low (LN) nitrogen fertilizer treatments. These 42 combinations of location, year and management will be called environments in the rest of the document. Experimental designs, estimation of adjusted means and heritabilities were presented in Rincent et al. (2019). Briefly summarized, in each environment the varieties were grouped in six to eight blocks according to their earliness. The designs were two-replicate designs or augmented designs. Adjusted means and heritabilities were estimated with SpATS (Rodríguez-Álvarez et al., 2018) for the two-replicate designs to take spatial trends into account and with block effect only for the augmented designs. On this dataset, the use of weights in the second step of the analysis to take into account the difference of precision of the adjusted means (Damesa et al., 2017) did not improve the results, and so a basic analysis without weights was applied here.

### Environmental Characterization and Estimation of an Environmental Covariance Matrix

Each environment was characterized by 139 environmental covariates (EC) in Rincent et al., 2019. Seventy-two of these covariates were estimated using climatic data (temperature, radiation), and 67 were estimated with SiriusQuality CGM (Martre et al., 2006) as dry matter stress index. These 139 covariates were estimated in each environment, transformed to standard normal distributions, and compiled in a matrix **Ω** of dimension (42 × 139).

**Ω** was then used to estimate a covariance matrix between environments (W). We applied the approach of Jarquín et al. (2014) in which all the ECs were used to estimate the covariance matrix. Environments with similar stresses are assumed to have similar GxE patterns. To compute *W*, we proceeded in two steps: first we computed the Euclidean distance matrix between environments (*D*_Ω_) with the matrix of environmental covariates (**Ω**), and then the covariance matrix *W* was computed as: $W=1_{E}-\frac{D_{\Omega }}{\text{max}\,\left(D_{\Omega }\right)}$. *W* does not take into account the targeted response variable, but the EC are supposed to be helpful to give an estimate of the true unknown environmental covariance matrix, as they reflect the conditions experienced by the plants. *W* was used in the downstream analysis as an estimate of the environmental covariance matrix.

### Selection of a Subset of Representative Environments

Because of the redundancy in the dataset due to strong similarities between the environments (Rincent et al., 2019), and to limit computational burden, a subset of sixteen environments was selected. The selection was applied in such a way that the different kinds of HD/GY relationship were explored. The 16 environments were composed of four environments with a low linear correlation between HD and GY (absolute value of the correlation <0.3), four environments with an intermediate HD/GY correlation (absolute value of the correlation between 0.3 and 0.6), four environments with a strong HD/GY correlation (absolute value of the correlation above 0.6), and four environments for which a quadratic relationship explained more than a linear relationship (Table 1). These four groups of environments were named “low,” “medium,” “high,” and “quadratic” according to the kind of relationship between HD and GY. The 16 environments were randomly sampled among the 42 environments of Rincent et al. (2019) with the constraint that each of the four groups of environments were represented by four environments. The prediction scenarios presented below were applied to these sixteen environments.

**TABLE 1:** Description of the 16 environments selected for the analysis.

Environment	Group^a^	Correlation HD/GY	$\hat{\alpha }$^b^	$\hat{\alpha }$^c^	$\hat{\beta }$^d^
Rea13WW	Low	–0.06	–0.34	–0.33	–0.32
Cle13LN	Low	0.19	0.14	0.10	–0.20
Sau13HN	Low	0.25	0.17	0.17	0.01
Gre12WW	Low	–0.28	–0.64	–0.64	–0.39
Mon12LN	Medium	0.50	0.81	0.83	–0.27
Cap12LN	Medium	0.51	0.59	0.68	–1.03
Gre13WD	Medium	–0.54	–0.82	–0.82	0.01
Mon13LN	Medium	0.59	1.03	1.03	–0.19
Vra13LN	High	0.61	0.73	0.74	0.06
Gre14WD	High	–0.64	–0.81	–0.82	–0.90
Gre12WD	High	–0.68	–0.99	–0.98	–0.39
Rec13LN	High	0.76	1.91	1.95	0.64
Cap12HN	Quadratic	0.38	0.18	0.36	–1.56
Coi12WW	Quadratic	0.34	0.50	0.18	–1.58
Lou12LN	Quadratic	0.35	0.82	0.39	–1.91
All14LN	Quadratic	0.19	0.20	0.19	–2.20

*^*a*^The sixteen environments were grouped in four classes according to the kind of relationship between HD and GY: “low,” “medium,” or “high” correlation, or “quadratic” relationship. ^*b*^α estimates in the 16 environments using model EG_HD. ^*c*^α and ^*d*^β estimates in the 16 environments using model EG_HD^2^. α and β correspond to the coefficients for the linear and quadratic regressions of GY on HD in each environment. The estimates of α and β presented here were obtained with the full dataset.*

### Prediction Objectives and the Corresponding Cross-Validation Schemes

Three prediction objectives were considered: the prediction of observed varieties in observed environments (oGoE) or in new environments (oGnE), and the prediction of new varieties in observed environments (nGoE). oGoE consists of predicting missing values in a MET (sparse testing), which typically corresponds to the situation faced by breeders when some observations are missing in their trial networks. oGnE and nGoE are more ambitious because predictions are made in an environment or for a variety without any phenotypic information on it.

To evaluate the performance of the prediction models in these three situations, three cross-validation schemes were defined: CVrandom, CVnewG, and CVnewE.

Observed varieties in observed environments was addressed by the CVrandom scheme, that is a 6-folds cross-validation, in which the folds were randomly sampled from the dataset. oGnE was addressed by the CVnewE scheme, which is a leave-one environment-out scheme, in which a new environment is predicted. nGoE was addressed by the CVnewG scheme, with a division in six folds consisting in five randomly selected groups of varieties.

Predictive abilities were computed for each fold and each environment as the correlation between adjusted means and predictions in the test set. For each cross-validation scheme except CVnewE (leave-one-out scheme), the total procedure was repeated 10 times to get robust estimates of predictive abilities.

### Reference Prediction Models

Three reference models with various levels of complexity were used to predict GY in the MET. In the first kind of models, the kinship matrix allowed sharing information between varieties, but there was no sharing of information between environments:

(1) $$\text{EG}:Y_{i\,j}=\mu_{j}+G_{i}+\in_{i\,j},\text{with}G_{i}∼N\left(0,K\sigma_{g\,1}^{2}\right)$$

(2) $$\text{EG\_GxE}:Y_{i\,j}=\mu_{j}+G_{i}+G\,E_{i\,j}+\in_{i\,j},\,\text{with}\,G\,E_{i\,j}∼N\,\left(0,K⊗I_{N_{E}}\,\sigma_{g\,2}^{2}\right)$$

*I*_*N_E*_ is an identity matrix of size the number of environments and ⊗ is the Kronecker product. μ_*j*_ is a fixed environmental effect, *G*_*i*_ is a random polygenic effect for variety *i*, and *GE*_*ij*_ is a random effect corresponding to the interaction between variety *i* and environment *j*. In all models, $\in_{i\,j}∼N\left(0,I\sigma_{e}^{2}\right)$, is the residual effect. In model EG the prediction of a variety is the same for any environment (main effect model). In model EG_GxE, GEI are taken into account, but there is no sharing of information between environments. Consequently, this second model is only applicable to scenarios CVrandom and CVnewG.

In the third model, information between environments is shared thanks to an environmental covariance matrix estimated with the environmental covariates as proposed by Jarquín et al. (2014):

(3) $$\text{EG\_GxW}:Y_{i\,j}=\mu_{j}+G_{i}+G\,E_{i\,j}+\in_{i\,j},\,\text{with}\,G\,E_{i\,j}∼N\,\left(0,K⊗W\,\sigma_{g\,3}^{2}\right)$$

W was estimated as described above.

### The Crop-Growth Modeling – Trait Assisted Prediction Model (CGM-TAP)

**FIGURE 1:**
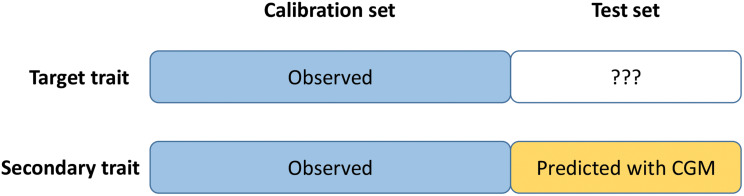
Schematic representation of CGM-TAP. CGM-TAP is a trait-assisted prediction approach in which the secondary trait is predicted using crop-growth modeling instead of being phenotyped.

Crop-growth modeling – trait assisted prediction is a trait assisted prediction model in which the secondary trait is observed for the calibration set but predicted for the test set using crop growth modelling (CGM) or gene-based crop-growth modelling (GBM) (Figure 1). The idea is to combine CGM and TAP to overcome the limit of GBM (not applicable yet to complex traits such as yield) and TAP (requires the phenotyping of the test set for the secondary trait in each environment). First (step 1), the CGM or GBM approach is applied to predict the secondary trait (HD) for the test set in each environment. Then (step 2), a trait-assisted prediction model is applied to the full dataset with the secondary trait as covariate (observed for the calibration set, predicted with CGM or GBM for the test set) with an environment specific effect to predict the target trait (GY) in each environment. In more details, CGM-TAP consists in the following two steps:

– Step 1: Prediction of the secondary trait (HD) for the test set using CGM. In this first step, the two main genetic parameters of the CGM involved in phenology (sensitivity to photoperiod “SLDL” and phyllochron “P”) are estimated (CVnewE and CVrandom) or predicted (CVnewG) for the test set. These variety specific genetic parameters are then used as input to the CGM together with the daily climatic data (temperature, daylength) to predict HD for the test varieties in each environment. In scenarios CVnewE and CVrandom, the test varieties are scored for HD in some environments, and so a Bayesian algorithm (Rincent et al., 2017) was used to directly estimate the genetic parameters of the test varieties. Briefly, the MCMC algorithm is a hybrid Gibbs sampler that updates the coordinates of the different parameters through a Metropolis-Hastings step using as proposal a Gaussian distribution centered on the previous value of the chain. In scenario CVnewG, there is no phenotypic information on the test varieties and so the genetic parameters cannot be estimated. So, a GBM approach was applied in which the genetic parameters are estimated for the calibration varieties, and then a GBLUP model (with major phenology markers as fixed effects) was used to predict the genetic parameters of the test varieties.
– Step 2: Different trait-assisted prediction models were then used to predict the target trait (GY) for the test set:

(4) $$\text{EG\_HD}:Y_{i\,j}=\mu_{j}+\alpha_{j}\times h_{i\,j}+G_{i}+\in_{i\,j},\,\text{with}\,G_{i}∼N\,\left(0,K\,\sigma_{g\,4}^{2}\right)$$

(5) $$\text{EG\_GxE\_HD}:Y_{i\,j}=\mu_{j}+\alpha_{j}\times h_{i\,j}+G_{i}+G\,E_{i\,j}+\in_{i\,j},\,\text{with}\,G\,E_{i\,j}∼N\,\left(0,K⊗I_{N_{E}}\,\sigma_{g\,5}^{2}\right)$$

(6) $$\text{EG\_GxW\_HD}:Y_{i\,j}=\mu_{j}+\alpha_{j}\times h_{i\,j}+G_{i}+G\,E_{i\,j}+\in_{i\,j},\,\mathrm{with}\,G\,E_{i\,j}∼N\,\left(0,K⊗W\,\sigma_{g\,6}^{2}\right)$$

with *h*_*ij*_ the heading date of variety *i* in environment *j*. *h*_*ij*_ was observed for the calibration varieties and predicted for the test set. α_*j*_is the effect of HD on GY in environment *j*.

Three other models similar to models 4–6 but with a quadratic effect of HD were also used:

(7) EG_HD:2Yi⁢j=μj+αj×hi⁢j+βj×hi⁢j2+Gi+εi⁢j

(8) EG_GxE_HD:2Yi⁢j=μj+αj×hi⁢j+βj×hi⁢j2+Gi+GEij+εi⁢j

(9) EG_GxW_HD:2Yi⁢j=μj+αj×hi⁢j+βj×hi⁢j2+Gi+GEij+εi⁢j

with $h_{i\,j}^{2}$ the squared heading date (centered and scaled) of variety *i* in environment *j*. β_*j*_ is the quadratic effect of HD on GY in environment *j*. All models are summarized in Table 2.

**TABLE 2:** Description of the reference and CGM-TAP models.

	Structure of the models				
	E	G	GEI	h	h^2^
**Reference models**					
EG	x	K	–	–	–
EG_GxE	x	K	*K*⊗*I_NE_*	–	–
EG_GxW	x	K	*K*⊗*W*	–	–
**CGM-TAP models**					
EG_HD	x	K	–	x	–
EG_GxE_HD	x	K	*K*⊗*I_NE_*	x	–
EG_GxW_HD	x	K	*K*⊗*W*	x	–
EG_HD^2^	x	K	–	x	x
EG_GxE_HD^2^	x	K	*K*⊗*I_NE_*	x	x
EG_GxW_HD^2^	x	K	*K*⊗*W*	x	x

*It is specified if the effect was included (x) or excluded (–) from the model. For mixed effects (G and GEI) the structure of the covariance matrix is indicated.*

For CVnewE, α_*j*_ and β_*j*_ cannot be estimated in models 4–9 because there is no observation of HD and GY in the test environment even for the calibration set, and so an alternative procedure was applied. First, α_*j*_ and β_*j*_ were estimated using a linear or a quadratic regression of GY on HD in each environment except the test environment. The estimates of the regression coefficients were then used to fit a multiple linear regression on the EC measured in these environments. A stepwise forward-backward procedure (function “step” of R package “stats” with a penalty *k* = 3.7 for α and *k* = 8 for β) was applied to select the most relevant EC and estimate their effects. This calibrated model was then used to predict α_*j*_ and β_*j*_ in the test environment. The estimates ${\hat{\alpha }}_{j}$ and ${\hat{\beta }}_{j}$ were plugged into models 4–9, which could then be used to predict GY in the test environment. We suppose here that the target environments are characterized by EC, so they are not totally unknown.

For each model, predictions were obtained as the sum of the BLUEs of the fixed effects and the BLUPs of the random effects. For each cross-validation scenario, the predictive ability (correlation between the predictions and the adjusted means) and root mean square error (RMSE) of HD in step 1 was estimated, as well as the RMSE of α_*j*_ and β_*j*_ for CVnewE. The predictive ability of GY in step 2 was evaluated for each model as presented above.

## Results

### Relationship Between HD and GY in the Sixteen Environments

**FIGURE 2:**
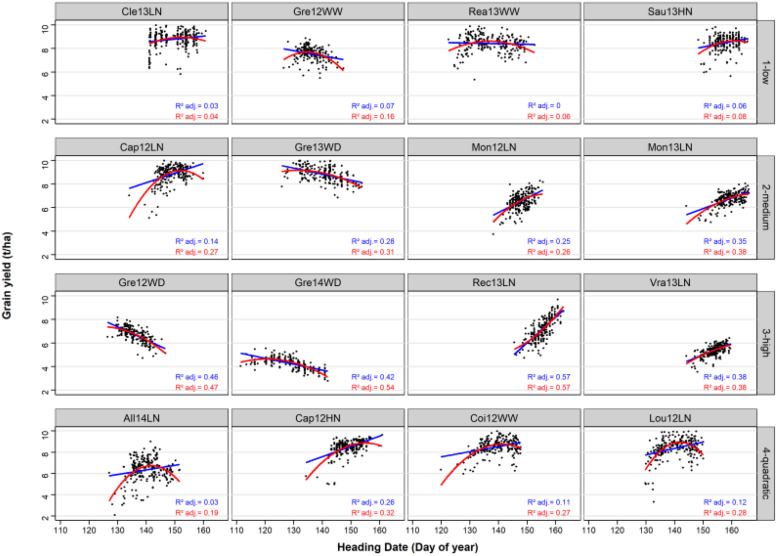
Linear and quadratic regressions of GY on HD in the 16 environments and the 220 bread wheat varieties. The linear and the quadratic adjustments are represented by a blue line and a red curve, respectively. Adjusted R^2^ are indicated for the linear (in blue) and for the quadratic (in red) adjustments.

The variance of HD was highly variable from one environment to another (Figure 2). HD was constraint to 15 days in Vra13LN whereas it covered around 30 days in other environments (Gre14WD, Coi12WW).

The average adjusted R^2^ of the linear relationship was of 0.04, 0.26, 0.46, and 0.13 in the low, medium, high and quadratic classes, respectively. For the quadratic environments, the adjusted *R*^2^ of the quadratic regression was of 0.27 on average, and higher than the adjusted *R*^2^ of the linear regression. In all four environments of this class, the quadratic relationship was concave with an optimal HD.

### Predictive Ability and RMSE of the Secondary Trait (HD) in the Different Scenarios

Before looking at the predictive abilities of GY with the different models, we looked at the predictive ability of the secondary trait (HD) in the different cross-validation scenarios (Table 3). The average predictive abilities of HD were above 0.78 in all scenarios, and it was higher in scenarios CVrandom and CVnewE (0.91) than in CVnewG (0.78). This was expected as the genetic parameters of the CGM had to be predicted in scenario CVnewG. RMSE was low in the three scenarios (average always below 3.33 days), and it was lower for CVrandom (1.86 days) than for CVnewG (2.88 days) and CVnewE (3.33 days). There was a strong variability of RMSE between folds with a maximum of 6.63 days in scenario CVnewE.

**TABLE 3:** Predictive ability and RMSE of the secondary trait (HD) in the three prediction scenarios.

	CVrandom			CVnewG			CVnewE		
	Min.	Mean	Max.	Min.	Mean	Max.	Min.	Mean	Max.
Pred. ability	0.68	0.91	0.98	0.49	0.78	0.95	0.86	0.91	0.96
RMSE (days)	0.81	1.86	3.40	1.39	2.88	5.37	1.62	3.33	6.63

*CVrandom corresponds to sparse testing, CVnewG to the prediction of unobserved varieties, and CVnewE to the prediction of unobserved environments. CVrandom and CVnewG were 6-folds cross-validations repeated ten times, and CVnewE was a leave-one-out scenario. Predictive abilities were computed as the correlation between adjusted means and predictions. RMSE are the root mean squared errors.*

### Prediction of the Regression Coefficients of HD on GY (α and β) in Scenario CVnewE

For models EG_HD, EG_GxE_HD, and EG_GxW_HD, it was necessary to first predict the value of α to be able to run the models in CVnewE, because no phenotypic observation is available on the predicted environment and thus, the relationship between HD and GY could not be directly estimated. For the same reasons, both α and β should be predicted before running models EG_HD^2^, EG_GxE_HD^2^, and EG_GxW_HD^2^.

**FIGURE 3:**
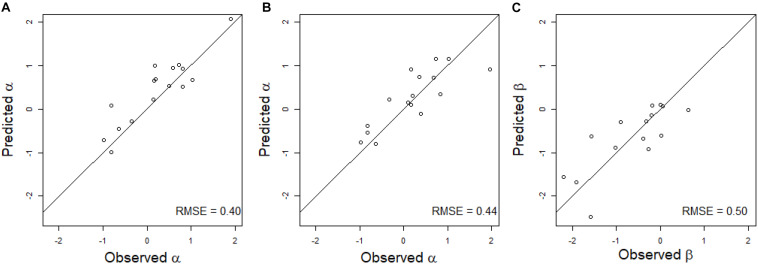
Scatter plot of the predicted and observed values of the regression coefficient of a linear regression **(A)** or of a quadratic regression **(B,C)** of GY on HD in scenario CVnewE. Observed α and β correspond to the estimates obtained from the linear **(A)** and quadratic **(B,C)** regressions of GY on HD in each environment. Predicted α and β correspond to the prediction of the α **(A)** or α and β **(B,C)** of the predicted environment (leave-one-environment-out scheme), using a multiple linear regression of the α **(A)** or α and β **(B,C)** estimated in the calibration environments on the environmental covariates. The black line corresponds to the line *y* = *x*.

The multiple linear regression on the environmental covariates was moderately efficient to predict the value of α for the linear regression of HD on GY in the new environment (Figure 3A). The RMSE was equal to 0.40 for observed α values varying between −0.99 and 1.91. For all environments except one, the environmental covariates were able to accurately predict the sign of the regression.

The predictions of α and β were less accurate for the quadratic linear regression of GY on HD, with RMSE equal to 0.44 for α, and 0.50 for β (Figures 3B,C). For β, the sign of the regression was inaccurately predicted for three environments.

### Predictive Ability of the Target Trait (GY) in the Different Scenarios

The predictive abilities for the target trait (GY) were highly variable between scenarios and between models (Table 4). On average over the four kinds of environments (low, medium, high and quadratic), the highest predictive abilities were obtained in scenario CVrandom (0.73 for EG_GxW_HDpred and EG_GxW_HDpred^2^). It was slightly lower for CVnewE (0.70 for EG_GxW_HDpred) and CVnewG (0.57 for EG_GxW_HDpred). The inclusion of GEI effects in the model (GxE and GxW) always increased predictive abilities in comparison to the main effect model EG, in particular when EC were used to estimate covariance between environments (GxW). On average over the four kinds of environments, the introduction of term GxW increased predictive abilities by 39% for scenario CVrandom, 37% for CVnewG, and 25% for CVnewE in comparison to the main effect model.

**TABLE 4:** Predictive abilities of the reference and of the CGM-TAP models in three prediction scenarios with HD as the secondary trait and GY as the target trait.

	CVrandom					CVnewG					CVnewE				
Models	Low	Medium	High	Quadratic	Average	Low	Medium	High	Quadratic	Average	Low	Medium	High	Quadratic	Average
EG	0.57	0.55	0.27	0.66	0.51	0.44	0.43	0.22	0.52	0.40	0.61	0.57	0.28	0.68	0.54
EG_HDpred	0.59	0.72	0.65	0.66	0.66	0.46	0.56	0.50	0.53	0.51	0.65	0.60	0.66	0.64	0.64
EG_HD^2^pred	0.59	0.71	0.66	0.68	0.66	0.41	0.55	0.48	0.40	0.46	0.64	0.62	0.66	0.14	0.51
EG_GxE	0.66	0.67	0.54	0.67	0.64	0.53	0.57	0.56	0.54	0.55	–	–	–	–	–
EG_GxE_HDpred	0.67	0.73	0.65	0.68	0.68	0.54	0.59	0.59	0.54	0.57	–	–	–	–	–
EG_GxE_HD^2^pred	0.66	0.71	0.64	0.70	0.68	0.50	0.59	0.54	0.45	0.52	–	–	–	–	–
EG_GxW	0.71	0.73	0.70	0.70	0.71	0.54	0.57	0.57	0.53	0.55	0.68	0.69	0.62	0.70	0.67
EG_GxW_HDpred	0.72	0.76	0.75	0.70	0.73	0.55	0.59	0.60	0.54	0.57	0.65	0.68	0.80	0.66	0.70
EG_GxW_HD^2^pred	0.71	0.75	0.74	0.72	0.73	0.52	0.59	0.55	0.46	0.53	0.60	0.73	0.78	0.24	0.59

*CVrandom is a 6-fold cross-validation scheme in which the test set was randomly sampled from the full dataset (sparse testing). CVnewG is a 6-fold cross-validation scheme, for which the objective is to predict new varieties in known environments. CVnewE is a leave-one environment-out scheme, in which the objective is to predict the performance of known varieties in a new environment. “low,” “medium,” “high,” and “quadratic” correspond to groups of four environments with different kinds of HD/GY relationships (Table 1 and Figure 2). No results are indicated for models involving the GxE term in scenario CVnewE, because these models are unable to make predictions in environments without any phenotypic data.*

For all scenarios and kinds of environment, the best model was always a CGM-TAP model, except in CVnewE for “low” and “quadratic” environments. The inclusion of HD as fixed effect (CGM-TAP) in the three reference models (EG, EG_GxE, and EG_GxW) always increased the average (over the four kinds of environments) predictive abilities. This increase strongly depended on the kind of environment and on the reference model considered. For the environment with a low correlation between GY and HD, and the environments with a quadratic relationship, the inclusion of HD as fixed effect in the reference models resulted in similar predictive abilities than those of the reference models, or to lower predictive abilities in CVnewE. Inclusion of HD increased predictive abilities of the reference models for environments with a medium GY/HD relationship. This increase was of 31, 30, and 5% in CVrandom, CVnewG and CVnewE, respectively, for model EG. As expected, the interest of CGM-TAP was highest for environments with a strong GY/HD relationship (“high”). For these environments the increase brought by CGM-TAP model was of 141, 127, and 132% in comparison to model EG in CVrandom, CVnewG and CVnewE, respectively. This increase was high but less pronounced for more complex reference models: it was of 7.1, 5.3, and 29.0% in comparison to model EG_GxW in CVrandom, CVnewG and CVnewE, respectively. Interestingly, in many cases, the simple CGM-TAP models (EG_HDpred, and ED_GxE_HDpred) performed as well as complex model involving environmental covariates (EG_GxW).

The introduction of a quadratic relationship in the reference models only slightly increased predictive abilities in scenario CVrandom. In other scenarios, the introduction of a quadratic relationship resulted in similar or lower abilities. It considerably decreased predictive abilities in scenario CVnewE, in which the regression coefficients (α and β) have to be predicted first using the EC (there is no phenotypic information in the test environments, and so the regression coefficients of the GY/HD relationship cannot be directly estimated).

## Discussion

In plant breeding, the knowledge that we have on the candidate varieties is limited because of the necessity to control phenotyping costs and the duration of the breeding cycles. This lack of information considerably limits the chance of identifying top varieties candidate for the registration process. Genomic prediction is a promising tool to screen early generation material (Heffner et al., 2010), because it allows transferring the information collected on the previous breeding cycles to other combination of genes and environmental conditions even in presence of GEI. Different models were proposed in the past years to predict GEI, which involved genetic and environmental characteristics (markers and EC) (Heslot et al., 2014; Jarquín et al., 2014; Cooper et al., 2016; Ly et al., 2018). We developed a new approach, called CGM-TAP, in which a secondary trait is predicted for the test set in each environment thanks to CGM. This secondary trait, easy to predict and capturing GEI, is then used as an environment specific covariate in usual GEI GS models. To sum up, it is a trait assisted prediction model, in which the secondary trait is predicted thanks to CGM instead of being phenotyped. Here we used HD and GY as secondary and target traits, respectively, to evaluate this new approach.

The results presented here reveal that in almost all situations, the CGM-TAP models performed at least as good as the corresponding reference models. It performed even better than the most sophisticated reference model involving EC (EG_GxW) with an increase of predictive ability of 7.1, 5.3, and 29.0% for the environments with the highest correlation between HD and GY (“high”) in scenarios CVrandom, CVnewG and CVnewE, respectively. The increase of predictive ability was generally stronger when the relationship between the secondary and the target trait was strong, as expected. But even for the “low” environments, CGM-TAP models (without the quadratic relationship) performed at least as good as the reference models. In the three cross-validation scenarios there were even situations in which the simple CGM-TAP model including only the main effects and the secondary trait (EG_HD) performed as good as the most sophisticated reference model EG_GxW (Table 4).

However, the introduction of a quadratic effect in the CGM-TAP models did not lead to a higher predictive ability and was even detrimental for the “quadratic” environments in scenario CVnewE. This is because we were unable to predict the regression coefficients α and β sufficiently well in new environments (Figure 3). The use of a larger set of environments would probably help to better predict these coefficients. Another option would be to use CGM to directly predict the HD/GY relationship in new environments, but we were unable to do it accurately. In other prediction scenarios (CVrandom and CVnewG), the introduction of the quadratic effect resulted in similar or lower predictive abilities in comparison to the CGM-TAP models without quadratic effect. This may be due to the necessity to reach more precise prediction of the secondary trait if a quadratic relationship is fitted. Another explanation is that the quadratic relationships were not very pronounced in this dataset (Figure 2).

These promising results show that the predictions of the secondary trait by the CGM were sufficiently accurate to be able to run a trait-assisted prediction model (without quadratic relationship) in all three prediction scenarios without phenotyping the test set. The RMSE of the prediction of HD with the CGM were indeed relatively low (Table 3). It was equal to 1.86 days in scenario CVrandom, which is close to usual field phenotyping error for this trait. The interest of the CGM-TAP models is dependent on our ability to predict the secondary trait. This means that CGM-TAP should work better if the calibration set was bigger, or was phenotyped in a MET optimized for the genetic parameters estimation (Rincent et al., 2017). Note that the use of the observed HD instead of the predicted HD as covariate in the CGM-TAP models resulted in much higher predictive abilities (results no shown). This means that CGM-TAP could work much better if we were able to predict HD more accurately.

Most studies on multi-trait and trait-assisted predictions are based on mixed models involving a genetic correlation between secondary and target traits (Rutkoski et al., 2016; Sun et al., 2017; Michel et al., 2018; Lado et al., 2018; Schulthess et al., 2018). Here, we proposed to introduce the secondary trait as covariate as in Crain et al. (2018), which allows modeling non-linear relationship between traits. This is useful in some situations with complex dependencies as illustrated by the quadratic relationship between HD and GY in some environments. This could also be the case with other secondary traits, for which there is an optimum value depending on the environment such as aerial or root biomass.

The choice of HD as a secondary trait was justified by the fact that it is easily predicted by CGM (Bogard et al., 2014), and it is an adaptive trait, and so is expected to be related to GY, at least in some environments (Richards, 1991; Semenov et al., 2014; Flohr et al., 2017). The relationship between phenology and productivity is important for most species (Andrade, 1995; Ouk et al., 2007), and so we can expect our strategy to be valuable for other crops. This relationship was confirmed in our dataset in which environments with strong HD/GY correlations or quadratic relationship could be found (Figure 2). However, the range of HD in breeding programs would be narrower and the correlation probably less pronounced. HD was a good candidate to test this approach, but other traits even more related to the target trait could be used. The important development of high-throughput phenotyping and of crop-growth modeling should make it possible to predict other yield related traits in the future. A suitable secondary trait to run CGM-TAP models should be accurately predicted with CGM and strongly related to the target trait. Traits related to light interception (LAI) or even yield components are secondary traits of choice and may be accurately predicted thanks to CGM in the near future with the help of HTP.

Note that the predictive abilities obtained in scenario CVnewG are probably higher in this study as would be in practice, as the folds of the cross-validation were randomly sampled. This indeed implies that varieties in the calibration set and in the test set are sampled from a same population, which is not the case in a real breeding scheme. Considering the reference models, the results of this study confirm that the introduction of an interaction (GEI) term in the prediction model increased predictive abilities in comparison to a main effect model for all scenarios. The model involving the environmental covariance matrix *W* (EG_GxW), performed better than model EG_GxE in scenario CVrandom, but similarly in scenario CVnewG. A major advantage of EG_GxW is that it allowed predicting in new environments, and these predictions were much more accurate than the main effect model. This is in agreement with previous publications (Jarquín et al., 2014; Malosetti et al., 2016; Rincent et al., 2019).

## Conclusion

In this paper, we propose a new way to predict genotype by environment interactions (CGM-TAP). This is a trait-assisted prediction approach in which the secondary trait is predicted by a crop growth model, instead of being phenotyped. The relationship between the secondary trait and the target trait is environment specific and thus allow predicting environment specific effects. This approach applied to yield (target trait) and heading date (secondary trait) increased predictive abilities of all reference GEI models, even those involving sophisticated environmental covariates, for various prediction scenarios (new varieties, new environments and sparse testing). This proof of concept could be applied to other traits in breeding programs in the near future, and is a new way of combining crop growth modeling and genomic prediction in the context of GEI.

## Data Availability Statement

The data supporting the conclusions of this article can be found at https://doi.org/10.15454/TKMGCQ.

## Author Contributions

RR defined the prediction strategy and prediction models, ran the analysis, and wrote the manuscript. PR ran the analysis and reviewed the manuscript. JL reviewed the manuscript, participated in the discussions, and contributed to the data production with the BreedWheat Consortium. All authors contributed to the article and approved the submitted version.

## Conflict of Interest

The authors declare that the research was conducted in the absence of any commercial or financial relationships that could be construed as a potential conflict of interest.

## References

[B1] Andrade F. H. (1995). Analysis of growth and yield of maize, sunflower and soybean grown at Balcarce, Argentina. *Field Crops Res.* 41 1–12. 10.1016/0378-4290(94)00107-N

[B2] Bogard M. Ravel C. Paux E. Bordes J. Balfourier F. Chapman S. C. (2014). Predictions of heading date in bread wheat (*Triticum aestivum* L.) using QTL-based parameters of an ecophysiological model. *J. Exp. Bot.* 65 5849–5865. 10.1093/jxb/eru328 25148833 PMC4203124

[B3] Burgueño J. de los Campos G. Weigel K. Crossa J. (2012). Genomic prediction of breeding values when modeling genotype × environment interaction using pedigree and dense molecular markers. *Crop Sci.* 52 707–719. 10.2135/cropsci2011.06.0299

[B4] Bustos-Korts D. Boer M. P. Malosetti M. Chapman S. Chenu K. Zheng B. (2019). Combining crop growth modeling and statistical genetic modeling to evaluate phenotyping strategies. *Front. Plant Sci.* 10:1491. 10.3389/fpls.2019.01491 31827479 PMC6890853

[B5] Calus M. P. Veerkamp R. F. (2011). Accuracy of multi-trait genomic selection using different methods. *Genet. Sel. Evol.* 43:26. 10.1186/1297-9686-43-26 21729282 PMC3146811

[B6] Charmet G. Tran L.-G. Auzanneau J. Rincent R. Bouchet S. (2020). BWGS: a R package for genomic selection and its application to a wheat breeding programme. *PLoS One* 15:e0232422. 10.1371/journal.pone.0232422 32240182 PMC7141418

[B7] Chenu K. Chapman S. C. Hammer G. L. Mclean G. Salah H. B. H. Tardieu F. (2008). Short-term responses of leaf growth rate to water deficit scale up to whole-plant and crop levels: an integrated modelling approach in maize. *Plant Cell Environ.* 31 378–391. 10.1111/j.1365-3040.2007.01772.x 18088328

[B8] Cooper M. Technow F. Messina C. Gho C. Totir L. R. (2016). Use of crop growth models with whole-genome prediction: application to a maize multienvironment trial. *Crop Sci.* 56 2141–2156. 10.2135/cropsci2015.08.0512

[B9] Crain J. Mondal S. Rutkoski J. Singh R. P. Poland J. (2018). Combining high-throughput phenotyping and genomic information to increase prediction and selection accuracy in wheat breeding. *Plant Genome* 11:UNS170043. 10.3835/plantgenome2017.05.0043 29505641 PMC12962554

[B10] Crossa J. de los Campos G. Maccaferri M. Tuberosa R. Burgueño J. Pérez-Rodríguez P. (2016). Extending the marker × environment interaction model for genomic-enabled prediction and genome-wide association analysis in durum wheat. *Crop Sci.* 56 2193–2209. 10.2135/cropsci2015.04.0260

[B11] Damesa T. Worku M. Möhring J. Piepho H. P. (2017). One step at a time: stage-wise analysis of a series of experiments. *Agron. J.* 109 845–857. 10.2134/agronj2016.07.0395

[B12] Fernandes S. B. Dias K. O. G. Ferreira D. F. Brown P. J. (2018). Efficiency of multi-trait, indirect, and trait-assisted genomic selection for improvement of biomass sorghum. *Theor. Appl. Genet.* 131 747–755. 10.1007/s00122-017-3033-y 29218378 PMC5814553

[B13] Flohr B. M. Hunt J. R. Kirkegaard J. A. Evans J. R. (2017). Water and temperature stress define the optimal flowering period for wheat in south-eastern Australia. *Field Crops Res.* 209 108–119. 10.1016/j.fcr.2017.04.012

[B14] Heffner E. L. Lorenz A. J. Jannink J. L. Sorrells M. E. (2010). Plant breeding with genomic selection: gain per unit time and cost. *Crop Sci.* 50 1681–1690. 10.2135/cropsci2009.11.0662

[B15] Henderson C. Quaas R. (1976). Multi-trait selection using relatives records. *J. Anim. Sci.* 43 218–218.

[B16] Heslot N. Akdemir D. Sorrells M. E. Jannink J.-L. (2014). Integrating environmental covariates and crop modeling into the genomic selection framework to predict genotype by environment interactions. *Theor. Appl. Genet.* 127 463–480. 10.1007/s00122-013-2231-5 24264761

[B17] Jarquín D. Crossa J. Lacaze X. Du Cheyron P. Daucourt J. Lorgeou J. (2014). A reaction norm model for genomic selection using high-dimensional genomic and environmental data. *Theor. Appl. Genet.* 127 595–607. 10.1007/s00122-013-2243-1 24337101 PMC3931944

[B18] Jia Y. Jannink J.-L. (2012). Multiple-trait genomic selection methods increase genetic value prediction accuracy. *Genetics* 192 1513–1522. 10.1534/genetics.112.144246 23086217 PMC3512156

[B19] Lado B. Vazquez D. Quincke M. Silva P. Aguilar I. Gutierrez L. (2018). Resource allocation optimization with multi-trait genomic prediction for bread wheat (*Triticum aestivum* L.) baking quality. *Theor. Appl. Genet.* 131 2719–2731. 10.1007/s00122-018-3186-3 30232499 PMC6244535

[B20] Lopes M. S. Saglam D. Ozdogan M. Reynolds M. (2014). Traits associated with winter wheat grain yield in Central and West Asia. *J. Integr. Plant Biol.* 56 673–683. 10.1111/jipb.12172 24456121

[B21] Ly D. Chenu K. Gauffreteau A. Rincent R. Huet S. Gouache D. (2017). Nitrogen nutrition index predicted by a crop model improves the genomic prediction of grain number for a bread wheat core collection. *Field Crops Res.* 214 331–340. 10.1016/j.fcr.2017.09.024

[B22] Ly D. Huet S. Gauffreteau A. Rincent R. Touzy G. Mini A. (2018). Whole-genome prediction of reaction norms to environmental stress in bread wheat (*Triticum aestivum* L.) by genomic random regression. *Field Crops Res.* 216 32–41. 10.1016/j.fcr.2017.08.020

[B23] Malosetti M. Bustos-Korts D. Boer M. P. van Eeuwijk F. A. (2016). Predicting responses in multiple environments: issues in relation to genotype × environment interactions. *Crop Sci.* 56 2210–2222. 10.2135/cropsci2015.05.0311

[B24] Martre P. Jamieson P. D. Semenov M. A. Zyskowski R. F. Porter J. R. Triboi E. (2006). Modelling protein content and composition in relation to crop nitrogen dynamics for wheat. *Eur. J. Agron.* 25 138–154. 10.1016/j.eja.2006.04.007

[B25] Messina C. D. Jones J. W. Boote K. J. Vallejos C. E. (2006). A gene-based model to simulate soybean development and yield responses to environment. *Crop Sci.* 46 456–466. 10.2135/cropsci2005.04-0372

[B26] Messina C. D. Technow F. Tang T. Totir R. Gho C. Cooper M. (2018). Leveraging biological insight and environmental variation to improve phenotypic prediction: integrating crop growth models (CGM) with whole genome prediction (WGP). *Eur. J. Agron.* 100 151–162. 10.1016/j.eja.2018.01.007

[B27] Meuwissen T. H. Hayes B. J. Goddard M. E. (2001). Prediction of total genetic value using genome-wide dense marker maps. *Genetics* 157 1819–1829. 11290733 10.1093/genetics/157.4.1819 PMC1461589

[B28] Michel S. Kummer C. Gallee M. Hellinger J. Ametz C. Akgöl B. (2018). Improving the baking quality of bread wheat by genomic selection in early generations. *Theor. Appl. Genet.* 131 477–493. 10.1007/s00122-017-2998-x 29063161 PMC5787228

[B29] Nakagawa H. Yamagishi J. Miyamoto N. Motoyama M. Yano M. Nemoto K. (2005). Flowering response of rice to photoperiod and temperature: a QTL analysis using a phenological model. *Theor. Appl. Genet.* 110 778–786. 10.1007/s00122-004-1905-4 15723276

[B30] Onogi A. Watanabe M. Mochizuki T. Hayashi T. Nakagawa H. Hasegawa T. (2016). Toward integration of genomic selection with crop modelling: the development of an integrated approach to predicting rice heading dates. *Theor. Appl. Genet.* 129 805–817. 10.1007/s00122-016-2667-5 26791836

[B31] Ouk M. Basnayake J. Tsubo M. Fukai S. Fischer K. S. Kang S. (2007). Genotype-by-environment interactions for grain yield associated with water availability at flowering in rainfed lowland rice. *Field Crops Res.* 101 145–154. 10.1016/j.fcr.2006.10.003

[B32] Prudent M. Lecomte A. Bouchet J.-P. Bertin N. Causse M. Genard M. (2011). Combining ecophysiological modelling and quantitative trait locus analysis to identify key elementary processes underlying tomato fruit sugar concentration. *J. Exp. Bot.* 62 907–919. 10.1093/jxb/erq318 21036926 PMC3022390

[B33] Pszczola M. Veerkamp R. F. de Haas Y. Wall E. Strabel T. Calus M. P. L. (2013). Effect of predictor traits on accuracy of genomic breeding values for feed intake based on a limited cow reference population. *Animal* 7 1759–1768. 10.1017/S175173111300150X 23915541

[B34] Purcell S. Neale B. Todd-Brown K. Thomas L. Ferreira M. A. R. Bender D. (2007). PLINK: a tool set for whole-genome association and population-based linkage analyses. *Am. J. Hum. Genet.* 81 559–575. 10.1086/519795 17701901 PMC1950838

[B35] Quilot B. Génard M. Lescourret F. Kervella J. (2005). Simulating genotypic variation of fruit quality in an advanced peach×*Prunus davidiana* cross. *J. Exp. Bot.* 56 3071–3081. 10.1093/jxb/eri304 16234284

[B36] Reymond M. Muller B. Leonardi A. Charcosset A. Tardieu F. (2003). Combining quantitative trait loci analysis and an ecophysiological model to analyze the genetic variability of the responses of maize leaf growth to temperature and water deficit. *Plant Physiol.* 131 664–675. 10.1104/pp.013839 12586890 PMC166842

[B37] Reymond M. Muller B. Tardieu F. (2004). Dealing with the genotype x environment interaction via a modelling approach: a comparison of QTLs of maize leaf length or width with QTLs of model parameters. *J. Exp. Bot.* 55 2461–2472. 10.1093/jxb/erh200 15286140

[B38] Richards R. (1991). Crop improvement for temperate Australia – future opportunities. *Field Crops Res.* 26 141–169. 10.1016/0378-4290(91)90033-R

[B39] Rimbert H. Darrier B. Navarro J. Kitt J. Choulet F. Leveugle M. (2018). High throughput SNP discovery and genotyping in hexaploid wheat. *PLoS One* 13:e0186329. 10.1371/journal.pone.0186329 29293495 PMC5749704

[B40] Rincent R. Charpentier J.-P. Faivre-Rampant P. Paux E. Le Gouis J. Bastien C. (2018). Phenomic selection is a low-cost and high-throughput method based on indirect predictions: proof of concept on wheat and poplar. *G3 (Bethesda)* 8 3961–3972. 10.1534/g3.118.200760 30373914 PMC6288839

[B41] Rincent R. Kuhn E. Monod H. Oury F.-X. Rousset M. Allard V. (2017). Optimization of multi-environment trials for genomic selection based on crop models. *Theor. Appl. Genet.* 130 1735–1752. 10.1007/s00122-017-2922-4 28540573 PMC5511605

[B42] Rincent R. Malosetti M. Ababaei B. Touzy G. Mini A. Bogard M. (2019). Using crop growth model stress covariates and AMMI decomposition to better predict genotype-by-environment interactions. *Theor. Appl. Genet.* 132 3399–3411. 10.1007/s00122-019-03432-y 31562567

[B43] Rodríguez-Álvarez M. X. Boer M. P. van Eeuwijk F. A. Eilers P. H. C. (2018). Correcting for spatial heterogeneity in plant breeding experiments with P-splines. *Spat. Stat.* 23 52–71. 10.1016/j.spasta.2017.10.003

[B44] Rutkoski J. Poland J. Mondal S. Autrique E. Pérez L. G. Crossa J. (2016). Canopy temperature and vegetation indices from high-throughput phenotyping improve accuracy of pedigree and genomic selection for grain yield in wheat. *G3 (Bethesda)* 6 2799–2808. 10.1534/g3.116.032888 27402362 PMC5015937

[B45] Schulthess A. W. Zhao Y. Longin C. F. H. Reif J. C. (2018). Advantages and limitations of multiple-trait genomic prediction for Fusarium head blight severity in hybrid wheat (*Triticum aestivum* L.). *Theor. Appl. Genet.* 131 685–701. 10.1007/s00122-017-3029-7 29198016

[B46] Schulz-Streeck T. Ogutu J. O. Gordillo A. Karaman Z. Knaak C. Piepho H.-P. (2013). Genomic selection allowing for marker-by-environment interaction. *Plant Breed.* 132 532–538. 10.1111/pbr.12105

[B47] Semenov M. A. Stratonovitch P. Alghabari F. Gooding M. J. (2014). Adapting wheat in Europe for climate change. *J. Cereal Sci.* 59 245–256. 10.1016/j.jcs.2014.01.006 24882934 PMC4026126

[B48] Sun J. Poland J. A. Mondal S. Crossa J. Juliana P. Singh R. P. (2019). High-throughput phenotyping platforms enhance genomic selection for wheat grain yield across populations and cycles in early stage. *Theor. Appl. Genet.* 132 1705–1720. 10.1007/s00122-019-03309-0 30778634

[B49] Sun J. Rutkoski J. E. Poland J. A. Crossa J. Jannink J.-L. Sorrells M. E. (2017). Multitrait, random regression, or simple repeatability model in high-throughput phenotyping data improve genomic prediction for wheat grain yield. *Plant Genome* 10:28724067. 10.3835/plantgenome2016.11.0111 28724067

[B50] Technow F. Messina C. D. Totir L. R. Cooper M. (2015). Integrating crop growth models with whole genome prediction through approximate bayesian computation. *PLoS One* 10:e0130855. 10.1371/journal.pone.0130855 26121133 PMC4488317

[B51] Touzy G. Rincent R. Bogard M. Lafarge S. Dubreuil P. Mini A. (2019). Using environmental clustering to identify specific drought tolerance QTLs in bread wheat (*T. aestivum* L.). *Theor. Appl. Genet.* 132 2859–2880. 10.1007/s00122-019-03393-2 31324929

[B52] Uptmoor R. Li J. Schrag T. Stützel H. (2011). Prediction of flowering time in *Brassica oleracea* using a quantitative trait loci-based phenology model: flowering time in *Brassica oleracea*. *Plant Biol.* 14 179–189. 10.1111/j.1438-8677.2011.00478.x 21973058

[B53] VanRaden P. M. (2008). Efficient methods to compute genomic predictions. *J. Dairy Sci.* 91 4414–4423. 10.3168/jds.2007-0980 18946147

[B54] White J. W. Herndl M. Hunt L. A. Payne T. S. Hoogenboom G. (2008). Simulation-based analysis of effects of Vrn and Ppd loci on flowering in wheat. *Crop Sci.* 48 678–687. 10.2135/cropsci2007.06.0318

[B55] White J. W. Hoogenboom G. (1996). Simulating effects of genes for physiological traits in a process-oriented crop model. *Agron. J.* 88 416–422. 10.2134/agronj1996.00021962008800030009x

[B56] Whittaker J. C. Thompson R. Denham M. C. (2000). Marker-assisted selection using ridge regression. *Genet. Res.* 75 249–252. 10.1017/S0016672399004462 10816982

[B57] Yin X. (2005). QTL analysis and QTL-based prediction of flowering phenology in recombinant inbred lines of barley. *J. Exp. Bot.* 56 967–976. 10.1093/jxb/eri090 15710636

[B58] Zheng B. Biddulph B. Li D. Kuchel H. Chapman S. (2013). Quantification of the effects of VRN1 and Ppd-D1 to predict spring wheat (*Triticum aestivum*) heading time across diverse environments. *J. Exp. Bot.* 64 3747–3761. 10.1093/jxb/ert209 23873997 PMC3745732

