# Supplementary material for: Combining Crop Growth Modeling With Trait-Assisted Prediction Improved the Prediction of Genotype by Environment Interactions
Source: Front Plant Sci. 2020 Jun 19;11:827. doi: 10.3389/fpls.2020.00827 (PMC7317015; doi:10.3389/fpls.2020.00827)
Supplement: Supplementary file 1 [file Table_1.docx]

Supplementary Table S1: Description of the 42 environments composing the multi-environment trial.

|  |  |  |  |  |  |  | **N fertilizer (kgN ha^-1^)** | **Generalized heritability** | |
| --- | --- | --- | --- | --- | --- | --- | --- | --- | --- |
| **Name^a^** | **Location** | **Altitude (m)** | **Longitude (°)** | **Latitude (°)** | **Treatment** | **Sowing** |  | **HD^b^** | **GY** |
|  |  |  |  |  |  | **date** |  |  |  |
| Cap12HN | Cappelle | 150 | 3.1 | 50.3 | HN | 10/28/2011 | 228 | 0.99 | 0.91 |
| Cap12LN | Cappelle | 150 | 3.1 | 50.3 | LN | 10/28/2011 | 130 | 0.99 | 0.92 |
| Cha12HN | Chalons | 102 | 4.4 | 48.95 | HN | 10/22/2011 | 200 | 0.93 | 0.89 |
| Cha12LN | Chalons | 102 | 4.4 | 48.95 | LN | 10/22/2011 | 100 | 0.91 | 0.9 |
| Coi12WW | Coings | 166 | 1.75 | 46.86 | HN | 10/17/2011 | 190 | 0.99 | 0.92 |
| Gre12WW | Gréoux | 300 | 5.86 | 43.75 | Irrigated | 11/17/2011 | 210 | 0.98 | 0.89 |
| Gre12WD | Gréoux | 300 | 5.86 | 43.75 | Dry | 11/17/2011 | 210 | 0.96 | 0.65 |
| Lou12HN | Louville | 144 | 1.82 | 48.19 | HN | 10/17/2011 | 150 | 0.97 | 0.93 |
| Lou12LN | Louville | 144 | 1.82 | 48.19 | LN | 10/17/2011 | 50 | 0.98 | 0.92 |
| Mau12HN | Maule | 54 | 1.85 | 48.91 | HN | 10/14/2011 | 180 | 0.98 | 0.95 |
| Mau12LN | Maule | 54 | 1.85 | 48.91 | LN | 10/14/2011 | 80 | 0.98 | 0.93 |
| Mon12HN | Mons | 85 | 3.01 | 49.88 | HN | 10/20/2011 | 200 | 0.97 | 0.81 |
| Mon12LN | Mons | 85 | 3.01 | 49.88 | LN | 10/20/2011 | 100 | 0.96 | 0.83 |
| Rec12HN | Réclainville | 150 | 1.75 | 48.34 | HN | 10/28/2011 | 150 | NA | 0.91 |
| Rec12LN | Réclainville | 150 | 1.75 | 48.34 | LN | 10/28/2011 | 50 | NA | 0.9 |
| All13HN | Allonnes | 150 | 1.65 | 48.34 | HN | 11/22/2012 | 201 | 0.98 | 0.94 |
| And13HN | Andelu | 97 | 1.69 | 48.86 | HN | 10/27/2012 | 240 | 0.99 | 0.89 |
| And13LN | Andelu | 97 | 1.69 | 48.86 | LN | 10/27/2012 | 160 | 0.99 | 0.9 |
| Cle13HN | Clermont-Ferrand | 331 | 3.15 | 45.46 | HN | 11/08/2012 | 250 | 0.98 | 0.86 |
| Cle13LN | Clermont-Ferrand | 331 | 3.15 | 45.46 | LN | 11/08/2012 | 175 | 0.88 | 0.9 |
| Fro13HN | Froissy | 160 | 2.22 | 49.57 | HN | 10/22/2012 | 180 | 0.99 | 0.69 |
| Gre13WD | Gréoux | 300 | 5.86 | 43.75 | Dry | 11/08/2012 | 225 | 0.96 | 0.6 |
| Lou13HN | Louville | 144 | 1.82 | 48.19 | HN | 11/02/2012 | 200 | 0.99 | 0.87 |
| Mau13HN | Maule | 54 | 1.85 | 48.91 | HN | 10/25/2012 | 170 | 0.98 | 0.96 |
| Mon13HN | Mons | 85 | 3.01 | 49.88 | HN | 10/24/2012 | 200 | 0.98 | 0.85 |
| Mon13LN | Mons | 85 | 3.01 | 49.88 | LN | 10/24/2012 | 100 | 0.99 | 0.86 |
| Rea13WW | Réalville | 100 | 1.5 | 44.14 | Irrigated | 11/14/2012 | 184 | 0.99 | 0.93 |
| Rec13LN | Réclainville | 150 | 1.75 | 48.34 | LN | 10/28/2012 | 100 | 0.99 | 0.88 |
| Sau13HN | Saultain | 70 | 3.58 | 50.34 | HN | 10/27/2012 | 185 | 0.86 | 0.5 |
| Ver13HN | Verneuil | 91 | 2.5 | 48.38 | HN | 10/29/2012 | 200 | 0.98 | 0.92 |
| Ver13LN | Verneuil | 91 | 2.5 | 48.38 | LN | 10/29/2012 | 100 | 0.96 | 0.89 |
| Vil13LN | Villier | 150 | 2.13 | 48.73 | LN | 10/23/2012 | 190 | 0.95 | 0.45 |
| Vra13HN | Vraux | 85 | 4.24 | 49.03 | HN | 10/23/2012 | 210 | 0.87 | 0.93 |
| Vra13LN | Vraux | 85 | 4.24 | 49.03 | LN | 10/23/2012 | 110 | 0.95 | 0.84 |
| All14HN | Allonnes | 150 | 1.65 | 48.34 | HN | 10/16/2013 | 157 | 0.97 | 0.89 |
| All14LN | Allonnes | 150 | 1.65 | 48.34 | LN | 10/16/2013 | 51 | 0.7 | 0.85 |
| Cha14WW | Champigny | 135 | 0.16 | 46.71 | Irrigated | 10/22/2013 | 180 | 0.99 | 0.90 |
| Cha14WD | Champigny | 135 | 0.16 | 46.71 | Dry | 10/22/2013 | 180 | 0.99 | 0.90 |
| Gre14WW | Gréoux | 300 | 5.86 | 43.75 | Irrigated | 11/07/2013 | 260 | 0.99 | 0.75 |
| Gre14WD | Gréoux | 300 | 5.86 | 43.75 | Dry | 11/07/2013 | 260 | 0.98 | 0.44 |
| Cle16WW | Clermont-Ferrand | 331 | 3.15 | 45.46 | Irrigated | 11/12/2015 | 70 | 0.94 | 0.78 |
| Cle16RO | Clermont-Ferrand | 331 | 3.15 | 45.46 | Dry (shelters) | 11/12/2015 | 140 | 0.94 | 0.75 |
| ^a^ HN, high N ; LN, low N ; WW, well-watered ; WD, water deficit ; RO, rainout shelter. | | | | | | | | |  |
| ^b^ NA, not available. | | | | | | | | |  |
